# Sudden Death in Young Competitive Athletes Due to Arrhythmogenic Cardiomyopathy: A 4-Decade National Referral Center Experience

**DOI:** 10.1161/CIRCEP.125.014877

**Published:** 2026-06-17

**Authors:** Monica De Gaspari, Kalliopi Pilichou, Alessandro Zorzi, Maria Bueno Marinas, Marco Cason, Rudy Celeghin, Patrizio Sarto, Ugo Fedeli, Ilaria Rigato, Alberto Cipriani, Martina Perazzolo Marra, Barbara Bauce, Domenico Corrado, Gaetano Thiene, Stefania Rizzo, Cristina Basso

**Affiliations:** 1 Cardiovascular Pathology Unit, Azienda Ospedaliera, Padua, Italy (M.D.G., K.P., M.B.M., M.C., R.C., S.R., C.B.).; 2 Department of Cardiac, Thoracic, Vascular Sciences and Public Health, University of Padua, Italy (M.D.G., K.P., A.Z., M.C., A.C., M.P.M., B.B., D.C., G.T., S.R., C.B.).; 3 Sports Medicine Unit, Regional Center for Exercise Prescription in Young Patients with Heart Diseases, ULSS2 Marca Trevigiana, Treviso, Italy (P.S.).; 4 Epidemiological Department, Azienda Zero, Veneto Region, Padova, Italy (U.F.).; 5 Cardiology Unit, Azienda Ospedaliera, Padua, Italy (I.R., A.C., M.P.M., B.B., D.C.).

**Keywords:** athletes, cardiomyopathies, exercise test, incidence, prevalence

## Abstract

**BACKGROUND:**

Arrhythmogenic cardiomyopathy (ACM) is a major cause of sudden cardiac death (SCD) in competitive athletes. We aimed to evaluate the prevalence and characteristics of ACM with the increased awareness of the disease at preparticipation screening after the introduction of the 1994 and 2010 diagnostic criteria.

**METHODS:**

The North-East Italy registry of juvenile SCD (≤40 years old) was searched for competitive athletes dying due to ACM in the time interval from 1985 to 2024. Cases referred from other regions were also included. Clinical and pathology data were analyzed according to guidelines.

**RESULTS:**

ACM was the cause of SCD in 29% of athletes. The incidence rate of SCD in athletes was 0.43 (0.27–0.65) versus 0.14 (0.05–0.33) per 100 000/y before and after 2010, respectively. Fifty-one athletes with ACM (50 men, 25±6.4 years) were enrolled. The pattern was right ventricular/biventricular in 74.5% and left ventricular in 25.5% (41% after 2010). Fibrofatty replacement was transmural in 63% of right ventricular/biventricular ACM and exclusively subepicardial-midmural in left ventricular ACM. First-line preparticipation screening revealed abnormalities in 62.7% (83.3% before 1994 and 40.9% after 2010). Twelve-lead ECG abnormalities were present in 50.9% (60.5% in right ventricular/biventricular ACM and 23% in left ventricular ACM), with negative T waves in 39.2% and low QRS voltage in 25.5%. Premature ventricular complexes/nonsustained ventricular tachycardia with left bundle-branch block or multiple morphologies were present on basal or limited exercise ECG in 35.3%. Maximal stress test, Holter, and 2-dimensional echocardiography were positive in 55%, 45.4%, and 5.8% of cases, respectively. In the only case who underwent contrast-enhanced cardiac magnetic resonance, late gadolinium enhancement was detected.

**CONCLUSIONS:**

ACM-related SCD incidence appeared lower in the post-2010 period. A phenotypic shift toward the left ventricular variant is observed, with ECG changes in a minority of cases and usually normal 2-dimensional echocardiography. If the index of suspicion is high, contrast-enhanced cardiac magnetic resonance is crucial for early identification and SCD prevention.

**WHAT IS KNOWN?**

- Mandatory preparticipation screening with a 12-lead ECG for competitive athletes in Italy has been shown to effectively identify individuals with cardiovascular diseases at risk of sudden death.
- The right ventricular variant of arrhythmogenic cardiomyopathy can be suspected in the majority of cases through resting 12-lead ECG during preparticipation screening followed by confirmatory testing using the diagnostic criteria.

**WHAT THE STUDY ADDS**

- Since the introduction of the 2010 diagnostic criteria and with the growing awareness of the disease, the prevalence of arrhythmogenic cardiomyopathy as a cause of sudden death among competitive athletes has declined, with a noticeable shift toward the left ventricular variant.
- The left ventricular arrhythmogenic cardiomyopathy variant is characterized by a low prevalence of electrocardiographic abnormalities and typically normal two-dimensional echocardiographic findings.
- A high index of suspicion is required in athletes with abnormalities at first-step preparticipation screening, particularly specific ECG changes such as low QRS voltages and ventricular arrhythmias on effort, prompting further investigations not limited to echocardiography but also including contrast-enhanced cardiac magnetic resonance.

Arrhythmogenic cardiomyopathy (ACM) is a genetically determined heart disease characterized by progressive myocyte death and substitution by fibrofatty or fibrous tissue of the ventricular myocardium.^1–3^ Initially considered a condition affecting only the right ventricle (RV; arrhythmogenic RV cardiomyopathy [ARVC]), ACM is now recognized as a disease involving both ventricles. Life-threatening ventricular arrhythmias (VAs) may occur during the course of the disease, particularly on effort, so that ACM is one of the leading causes of sudden cardiac death (SCD) in the competitive athletes.^4–8^ Early identification of affected athletes by preparticipation screening (PPS) with sports disqualification is life-saving. Up to the early 1990s athletes who died of ACM often had a history of syncopal episodes, ECG abnormalities consisting of inverted T waves in the right precordial leads, and VAs with a left bundle-branch block pattern.^4–8^ Nonetheless, they were not identified at PPS, because this disease was not widely recognized as a cause of SCD during sports activity and the criteria were neither available nor implemented. With the introduction of the 1994^9^ and 2010^10^ updated ARVC diagnostic criteria, there has been an increased awareness of the disease in the medical community, including sports physicians, leading to better identification at PPS. However, disease variants with left ventricular (LV) involvement are difficult to diagnose, unless contrast-enhanced cardiac magnetic resonance (CE-CMR) is performed.^11–14^

The present study aimed to: (1) assess the temporal trend of ACM variants as a cause of SCD over a 4-decade period; (2) identify alarming symptoms/signs at PPS through clinic-pathological correlations in affected athletes who died suddenly; and (3) evaluate the prevalence of ACM in the population-based Registry of SCD in young athletes of North-East Italy.

## Methods

The study complies with the Declaration of Helsinki. The gross and histological samples were used in accordance with the Recommendation CM/Rec (2016) 6 of the Committee of Ministers to Member States on research on biological materials of human origin, released by the Council of Europe, as received by the Italian National Council of Bioethics. The study was approved by the institutional review board, and no informed consent was required. The data that support the findings of this study are available from the corresponding author on reasonable request.

The Cardiovascular Pathology Unit, University Hospital, Padua, Italy, is a referral center for the study of juvenile SCD (people aged ≤40 years). The database was been searched for cases occurring in competitive athletes identified as affected by ACM at cardiovascular pathology examination in the time interval from 1985 to 2024.

According to the Regional Health System, every case of SCD occurring in young people in the Veneto Region, North-East Italy, should undergo autopsy, and the heart is referred to our Unit which works as a core laboratory for the morphological and genetic study.

To evaluate the proportion of SCD attributed to ACM, an analysis of causes of SCD among young athletes dying suddenly in the Veneto Region, where a registry of SCD in young people (<35 years old) has been established in 1985, was performed. The Veneto Region covers an area of 18 368 km^2^. The population is 4 849  553, according to the Italian Census Bureau 2022. For this purpose, to compare the results of the present study with the prevalence data previously published in 2006, the same age-range population was selected (12–35 years old).^6^

An analysis of causes of SCD among the young (aged 12–35 years) nonathletic population dying suddenly in the Veneto Region during the same study period was performed to compare ARVC/ACM between athletic and nonathletic populations.

In addition, to better assess the temporal trend of morphological variants of ACM as a cause of SCD in competitive athletes, we extended the analysis to include all cases of SCD in athletes, also encompassing those aged >35 up to 40 years and those referred from other geographic regions.

Former athletes who were disqualified and suffered SCD a few months later (<12 months) and were referred to our pathology core laboratory were also investigated.

According to the Italian guidelines for sport activity, competitive athletes are defined as people who participate in an organized sports program requiring regular training and competition.^15^ For these people, annual PPS has been required by law since 1982.

The sport considered for each athlete was identified based on the annual medical certificate of sport eligibility, as required by Italian law. Although some athletes may participate in multiple sports, the certification process requires the physician to indicate the primary sport for which the athlete undergoes eligibility assessment.

Sudden death is defined as unexpected death as a result of natural causes in which a loss of all functions occurs instantaneously or within 1 hour of the onset of collapse symptoms.

All cases were considered cardiac in origin after exclusion of noncardiac causes, including a negative toxicology screening for illicit or prescribed drugs.

The Strengthening the Reporting of Observational Studies in Epidemiology guidelines were used to ensure the reporting of this observational study.^16^

### Morphological Protocol

The protocol for the investigation of SCD and heart examination is the 1 reported in detail by the Association for European Cardiovascular Pathology.^17^ Macroscopic evaluation includes measurement of heart weight and wall thickness, inspection of the coronary arteries and valves, and identification of any myocardial scar or dilation. The origin and course of the coronary arteries were examined, and the patency of the major epicardial coronary arteries was analyzed by transverse sections at 3-mm intervals. A complete transverse (short-axis) cut of the heart at the midventricular level and then further parallel transverse slices of the ventricles at 1-cm intervals toward the apex were routinely performed. The slices were carefully inspected for changes in the cut surface of the myocardium.

Coronary artery segments and several transmural blocks of ordinary myocardium from a representative transverse slice of the ventricles to include the free wall of the LV (anterior, lateral, and posterior), the ventricular septum (anterior and posterior), and the free wall of the RV (anterior, lateral, and posterior), as well as the RV outflow tract, were taken for histological examination; 7-μm-thick sections were stained with the hematoxylin and eosin and trichrome Heidenhain (azan) techniques. In the setting of normal histology, a second round of blocks was taken from a parallel short-axis section of the heart before discharging the heart as normal.

The diagnosis of ACM is made at autopsy in the presence of gross or histological evidence of regional or diffuse fibrofatty or fibrous replacement of the ventricular myocardium with either a transmural distribution or confinement to the subepicardial and midmural layers, in the absence of coronary artery disease and other known cardiac or systemic diseases affecting the myocardium.^1,3,11,13^ A typical RV ACM variant is diagnosed in the presence of RV free wall fibrofatty replacement, with or without transmural involvement, possibly associated with LV free wall involvement (biventricular [BIV]-ACM). In the presence of isolated LV free wall fibrous or fibrofatty replacement with nonischemic distribution, a LV ACM variant is identified (also reported as a nonischemic LV scar). A subepicardial and midmural confluent area (at least 2 tissue blocks) of loose fibrous tissue repair with lymphocytic inflammatory infiltrates is categorized as an early ACM variant (hot phase).^1,3,11,13^

### Clinical Correlations

Clinical symptoms and signs were noted as reported at the time of PPS during personal history collection. In Italy, PPS for competitive sports is uniform across all regions, as it is mandated by national law and conducted according to the Cardiological Guidelines for Competitive Sports Eligibility, which are periodically revised and updated.^18,19^

As required by the Italian law,^15^ the athletic population has to undergo PPS by history, physical examination, 12-lead ECG, and limited exercise testing.^18,19^ Further examinations are prescribed by the sports medicine physician performing the PPS in cases of abnormalities on first-line tests. All examinations performed at PPS were carefully reviewed, including first-line (basal 12-lead ECG, screening exercise test; available in 100%) and second-step tests (ie, maximal stress-test ECG, 24-hour Holter monitoring, 2-dimensional [2D] echocardiography, and CE-CMR when available). Low QRS voltages (LQRSV) are here defined as a QRS amplitude from peak to nadir <0.5 mV in all limb leads on a basal 12-lead ECG. Holter monitoring is considered positive for VAs if it shows frequent/complex premature ventricular complexes (>500 per 24 hours), nonsustained or sustained ventricular tachycardia; a positive maximal stress test indicates VAs induced by exercise; and a positive 2D echocardiogram is defined by global or regional structural or functional RV abnormalities consistent with ARVC diagnostic criteria.

Information regarding the availability of an automated external defibrillator (AED) at the site of SCD was obtained through a detailed reconstruction of the event circumstances. Specifically, data were collected from medical records and emergency medical service reports, which allowed documentation of both the context of the event and the characteristics of the rescue intervention, including the presence or absence of an on-site AED.

### Statistical Analysis

Continuous variables were summarized as medians and interquartile ranges, whereas categorical variables were reported as counts and percentages. Differences in categorical variables were assessed using Fisher exact contingency test. Multiple-group comparisons were analyzed using the Kruskal-Wallis test, because of the small number of samples in each group and because the data did not follow a normal distribution. A value of *P*<0.05 was considered statistically significant. Incidence rates of SCD in athletes and nonathletes were calculated based on the population census of the Veneto Region: the average number of residents aged 12 to 35 years was ≈1 478 000 in the pre-2010 period and 1 180 000 in the post-2010 period. Based on the available data,^20,21^ we estimated that the number of individuals aged 12 to 35 years engaged in competitive sports was about 200 000 up to 2010 and 220 000 after 2010.

This slight increase reflects a rise in the proportion of young people participating in competitive sports, despite the overall decline in the youth population. Nonathletes were calculated as total residents minus athletes.

## Results

As a referral center for the study of SCD in the young, an overall population of 51 competitive athletes (50 men; age range, 14–40 years, mean age, 24.6±6.4 years) with ACM diagnosed at autopsy was collected and was available for clinic-pathological correlations. The most frequently practiced sport was soccer (23, 45.1%), followed by cycling (7, 13.7%), basketball (5, 9.8%), rugby (3, 5.8%), swimming (2, 3.9%), running (2, 3.9%), and bodybuilding, canoeing, golfing, hockey, martial arts, motocross, rowing, sports dance, and tennis (1 each, 1.9%). SCD occurred on effort in 34 (66.7%) cases, 23 of them during competition (45.1%) and 10 during training (19.6%). An AED was available on site in only 3 (5.9%) cases.

### Temporal Trends in Phenotypic Variants and PPS Results

Main clinical and pathological data are reported in Table 1. Thirty-eight (74.5%) were typical RV/BIV variants of ACM and 13 (25.5%) were LV ACM variants; 5 of them (9.8%) showed diffuse segmental lymphocytic myocarditis associated with to loose connective tissue repair (early ACM). In terms of transmurality, two thirds of RV/BIV variants showed transmural fibrofatty replacement in at least 1 ventricular wall tissue sample, whereas the LV ACM variants always had either subepicardial or subepicardial-midmural involvement. Genetic data are reported as supplemental information (Supplemental Material).

**Table 1.**
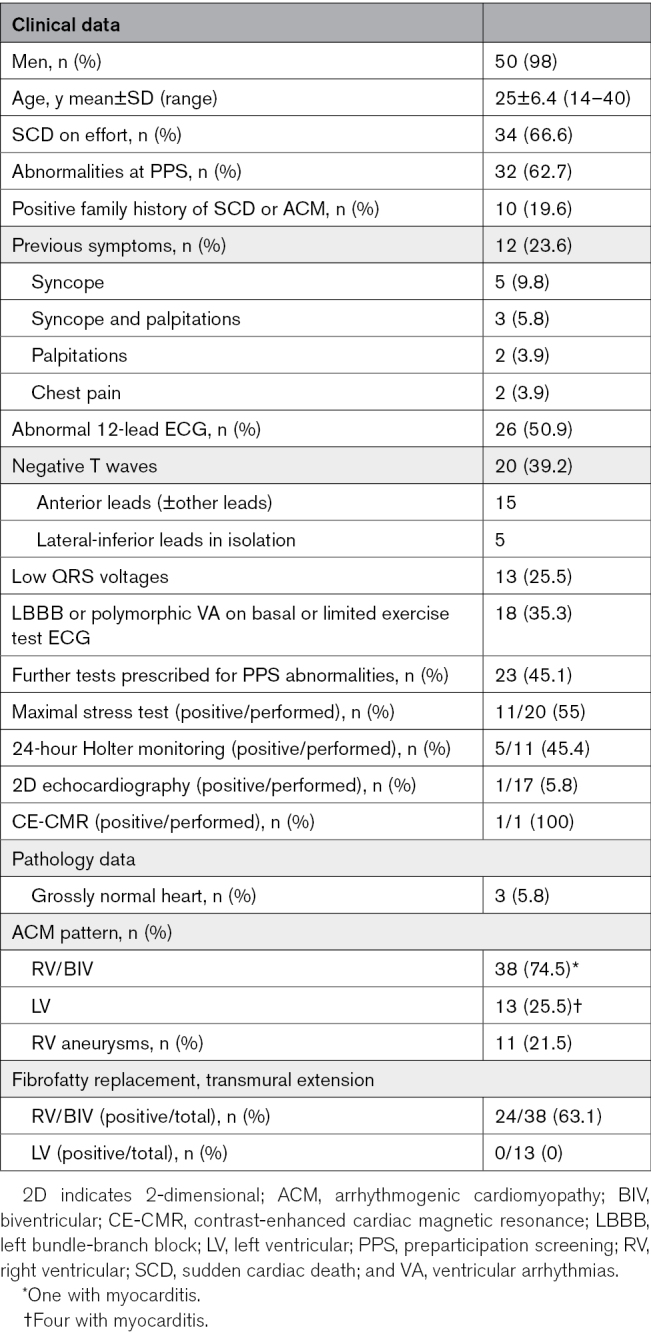
Main Clinical and Pathological Data in 51 Athletes With SCD Due to ACM

A subanalysis of PPS data and pathology data in 3 different eras, that is, before the introduction of the 1994 ARVC diagnostic criteria, between 1995 and 2010 (introduction of updated ARVC criteria), and after the 2010 ARVC criteria, is reported in Table 2.

**Table 2.**
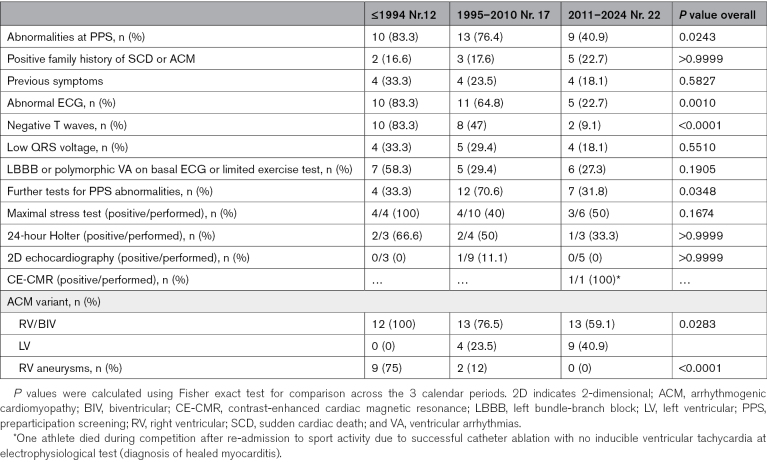
Analysis of PPS Data and ACM Variant by Study Period, Before and After the Introduction of 1994 Diagnostic Criteria and After the 2010 Updated Diagnostic Criteria

A subanalysis of PPS data by ACM variant is reported in Table 3.

**Table 3.**
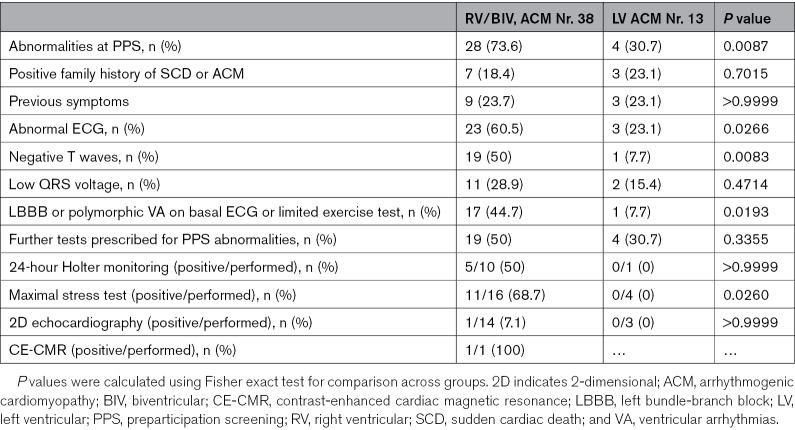
Analysis of PPS Data by ACM Variant Detected at Autopsy

**Figure 1.**
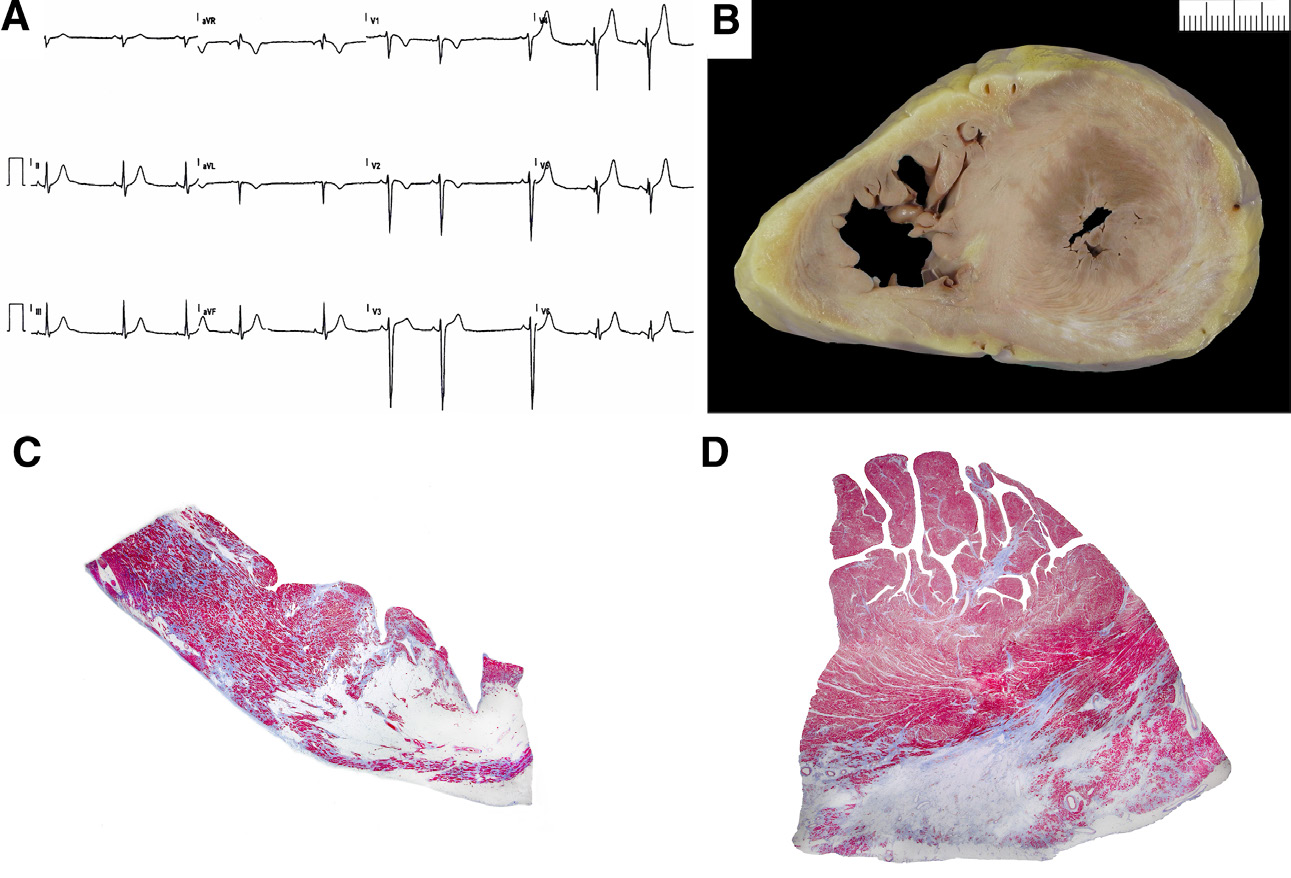
**A 16-year-old asymptomatic basketball player who died suddenly on the field. A**, Resting 12-lead ECG showing sinus arrhythmia, fragmented QRS complexes in V4–V6, delayed precordial S/R transition, and T-wave inversion in V1 and V2. **B**, Cross section of the heart showing subepicardial scarring in the left ventricular (LV) wall and yellow transmural discoloration of the right ventricular (RV) posterior wall, without thinning. **C**, Panoramic histological section of the RV posterior wall with segmental transmural fibrofatty replacement. **D**, Panoramic histological section of the LV posterolateral wall with subepicardial-midmural fibrofatty replacement.

**Figure 2.**
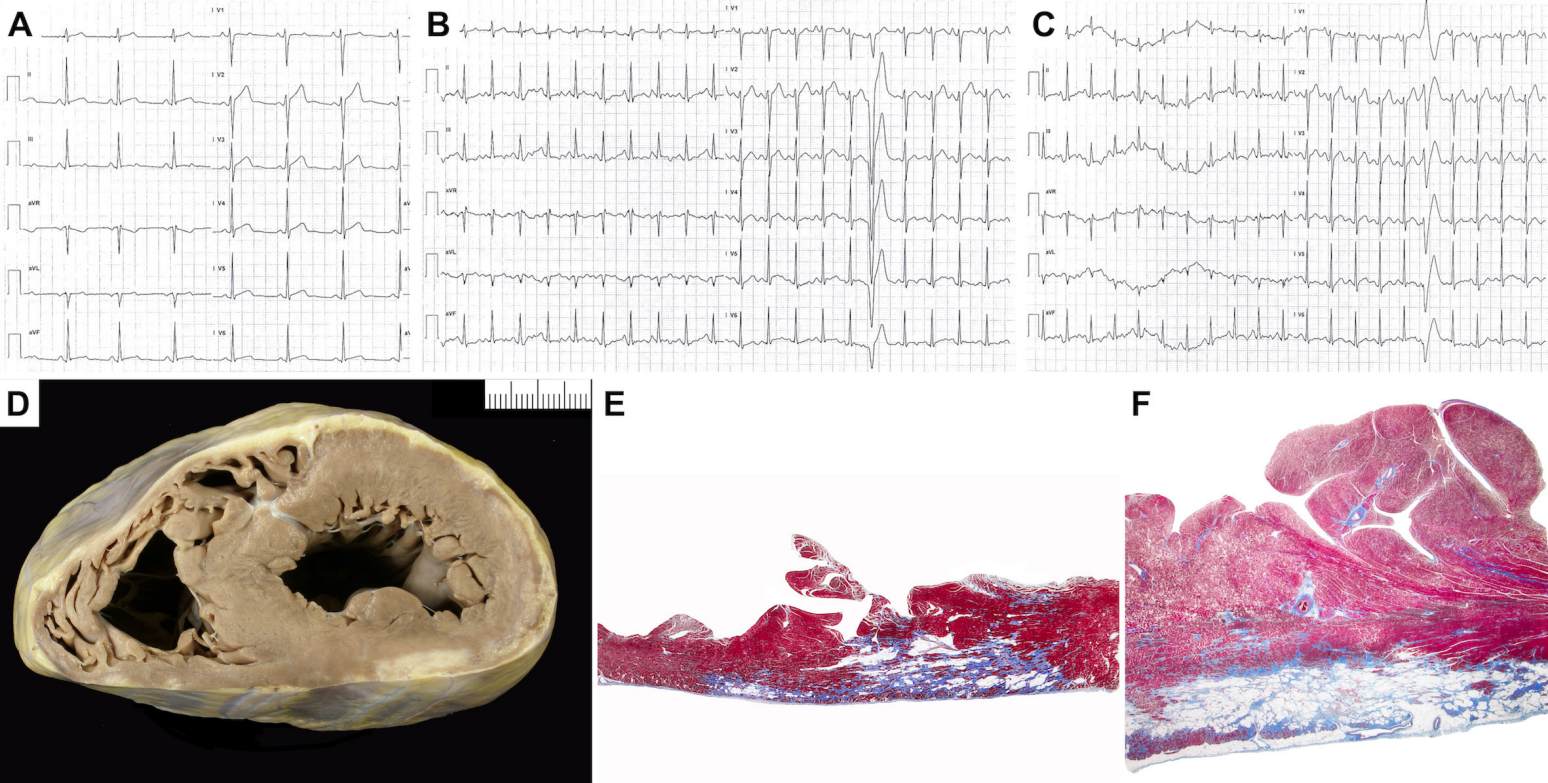
**A 26-year-old asymptomatic hockey player who died suddenly on the field. A**, Normal resting 12-lead ECG. **B**, Premature ventricular beat recorded during exercise testing, with a left bundle-branch block-like morphology. **C**, Premature ventricular beat recorded during exercise testing, with a right bundle-branch block-like morphology. **D**, Cross section of the heart showing subepicardial scarring in both the posterior left ventricular (LV) and right ventricular (RV) walls, in the absence of wall thinning. **E**, Panoramic histological section of the RV posterior wall with segmental fibrofatty replacement. **F**, Panoramic histological section of the LV posterior wall with subepicardial-midmural fibrofatty replacement.

**Figure 3.**
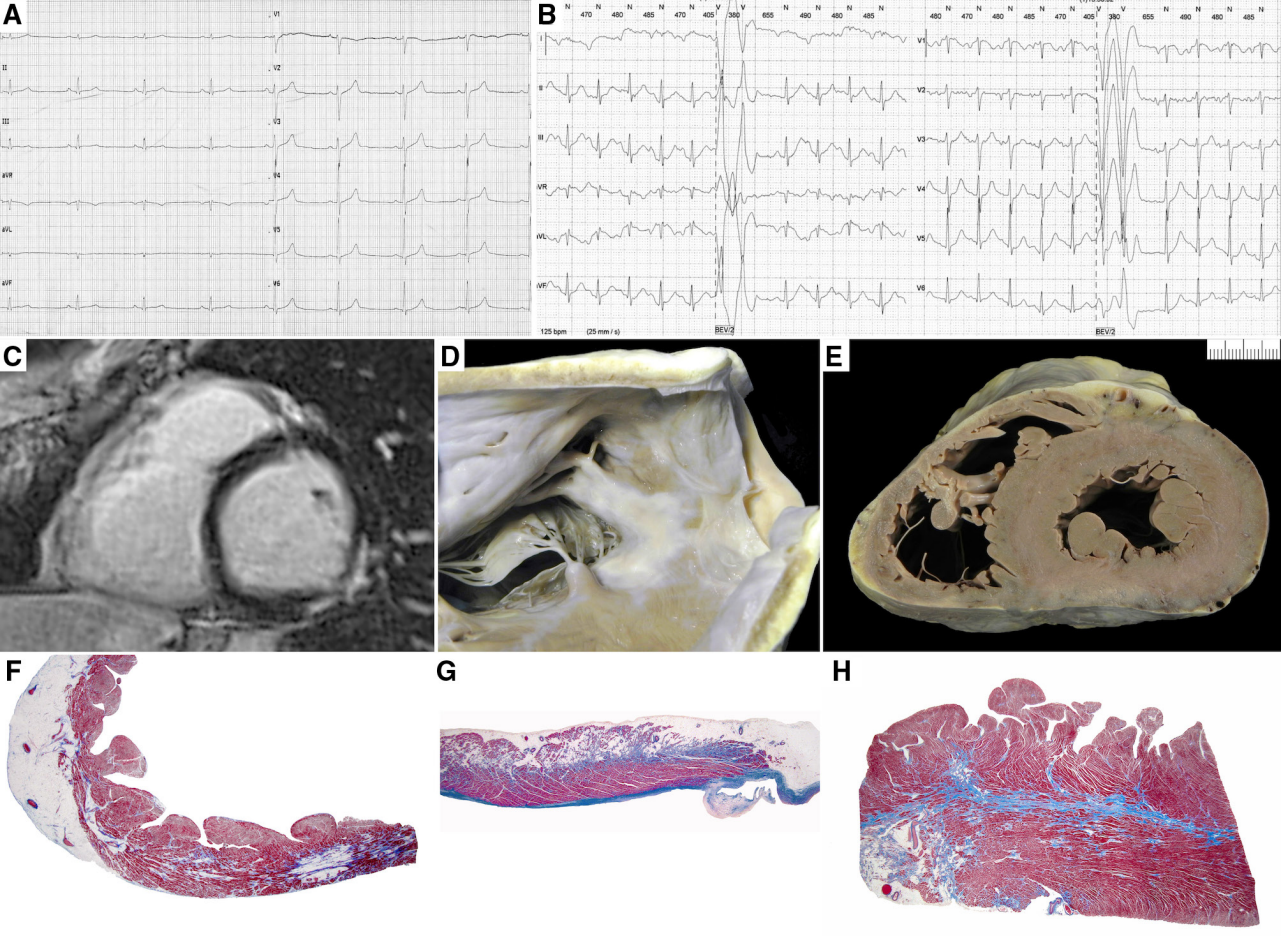
**A 40-year-old cyclist who underwent successful catheter ablation of a misdiagnosed idiopathic right ventricular (RV) outflow tract tachycardia and died suddenly during competition after return-to-play. A**, Resting 12-lead ECG showing low QRS voltages in the limb leads. **B**, Ventricular couplet recorded by ambulatory ECG monitoring during a training session (cycling), with a left bundle-branch block/inferior axis morphology, suggesting an origin from the RV outflow tract. **C**, Contrast-enhanced cardiac magnetic resonance showing focal posterolateral late gadolinium enhancement interpreted as healed myocarditis. **D**, At postmortem examination, the RV outflow tract shows the sequelae of catheter ablation with endocardial white fibrous thickening. **E**, Cross section of the heart without relevant macroscopic abnormalities. **F**, Panoramic histological section of the RV posterior wall with segmental fibrofatty replacement. **G**, Panoramic histological section of the RV outflow tract wall with subepicardial fibrofatty replacement. Note the whitish fibrous thickening of the endocardium after ablation. **H**, Panoramic histological section of the LV posterior wall with midmural fibrous scarring.

In 32 (62.7%) cases, first-line tests, including history and 12-lead ECG findings at PPS, revealed some abnormalities, whereas they were completely negative in 19 (37.3%) cases (Figures 1 and 2). Additional investigations were prescribed in 23 (45.1%) cases and consisted of maximal exercise testing in 20, 2D echocardiography in 17, 24-hour Holter monitoring in 11 and CE-CMR in 1. Two-dimensional echocardiography was positive in only 1 of 17 (5.8%) cases, whereas LV late gadolinium enhancement (LGE) was detected in the only athlete who underwent CE-CMR. The latter underwent successful catheter ablation of a misdiagnosed idiopathic RV outflow tract tachycardia, and the LV LGE was considered the result of previous myocarditis; he returned to play and died suddenly during a competition (Figure 3).

### Sudden Death of Former Athletes Not Eligible for Competitive Sport

Noteworthy, we studied 4 additional ex-athletes who were disqualified a few months before SCD (range, 6–12 months; mean, 8 months) from competitive sports activity due to PPS abnormalities, either temporarily (n=2, to be followed up after detraining) or permanently (n=2). Three died on effort during noncompetitive training. All had 12-lead ECG abnormalities at PPS (inverted T waves in the precordial leads in 4 and LQRSV in 3) and polymorphic VA during maximal stress testing. In all cases, 2D echocardiography was normal, whereas CE-CMR was positive for LV LGE. At autopsy, heart examination revealed a BIV variant of ACM.

### Prevalence of ACM as a Cause of SCD in Competitive Athletes in North-East Italy

In a consecutive series of 94 competitive athletes (86 males; age range, 12–35 years; mean age, 26.2±9.4 years) who suffered SCD in the Veneto Region, North-East Italy, ACM (including RV/BIV and LV variants) was identified at autopsy in 27 (28.7%). The remaining cases had atherosclerotic coronary artery disease (13, 13.6%), myocarditis (8, 8.4%), congenital coronary artery anomalies (7, 7.4%), hypertrophic cardiomyopathy (3, 3.1%), preexcitation syndrome (3, 3.1%), mitral valve prolapse (2, 2.1%), aortic dissection (2, 2.1%), dilated cardiomyopathy (1,1.0%), atrioventricular block (1, 1.0%), and 27 had a structurally normal heart (so-called unexplained SCD, 28.4%). When comparing the causes of SCD in young athletes in the same geographic area in 2 periods, that is, before and after the introduction of the updated 2010 ARVC diagnostic criteria, the prevalence of ACM decreased from 30.5% to 22.7%, with RV/BIV ACM accounting for 23.6% before 2010 versus 4.5% after 2010, and the LV ACM variant accounting for 6.9% before 2010 versus 18.2% after 2010.

### Causes of SCD in Competitive Athletes Versus Nonathletes in the Veneto Region, Italy

The causes of death in a consecutive series of 495 (345 males, 69.7%; age range, 12–35 years; mean age, 26.0±6.6 years) young nonathletic SCD victims in the same geographic area and time interval are reported in Table 4.

**Table 4.**
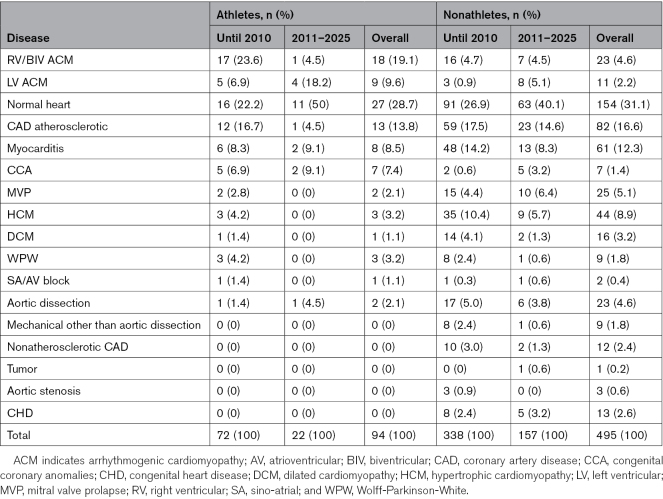
Causes of Sudden Cardiac Death in Young Athletes Versus Nonathletes, Veneto Region, Italy

The incidence rate of SCD in athletes per 100 000/y for every cause was 1.41 (95% CI, 1.10–1.78) versus 0.64 (95% CI, 0.40–0.97) before and after 2010, respectively. The incidence rate of SCD in nonathletes was 1.06 (95% CI, 0.95–1.18) versus 1.10 (95% CI, 0.94–1.29) in the same study periods.

The incidence rate of SCD in athletes per 100 000/y for ACM was 0.43 (95% CI, 0.27–0.65) versus 0.14 (95% CI, 0.05–0.33) before and after 2010, respectively. The incidence rate of SCD in nonathletes was 0.06 (95% CI, 0.04–0.09) versus 0.11 (95% CI, 0.06–0.19) in the same study periods.

## Discussion

Systematic PPS of young competitive athletes, which has been in practice in Italy for >40 years, has successfully prevented SCD from many cardiovascular diseases, including hypertrophic cardiomyopathy, through the identification of affected athletes and remodulation of exercise activity based on risk stratification.^6,7,22^ While other cardiovascular diseases are progressively decreasing in prevalence as a cause of SCD in competitive athletes, the current study shows that ACM remains the leading cause accounting for 29% of cases. A decrease is evident when analyzing the two study periods, that is, before and after the introduction of the 2010 updated ARVC diagnostic criteria, with ACM accounting for 31% of SCD in athletes in the former period versus 22% in the latter. A phenotypic shift of ACM at autopsy has been observed when comparing the pre- and post-2010 periods, with the RV/BIV variant decreasing from 26.4% to 13.6%, and the LV variant increasing from 4.2% to 9.1%, respectively.

The 12-lead ECG is not informative in 3 quarters of LV ACM variants, and 2D echocardiography shows mostly normal or borderline findings.

Rather than demonstrating a policy effect, the main contribution of this study lies in documenting a progressive phenotypic shift of ACM toward LV variants, as confirmed by systematic postmortem examination. This observation provides a pathophysiological explanation for the reduced sensitivity of ECG and echocardiography in contemporary cases and highlights the growing importance of CE-CMR in athletes with suspected disease.

### Characteristics of Athletes Dying of ACM

We previously demonstrated that athletes who died of ACM in the 1990s often had a history of syncopal episodes, ECG changes, and VAs with a left bundle-branch block pattern, but they escaped the identification at PPS because of low awareness of the disease as a cause of SCD during sports activity and because the criteria were neither available nor implemented.^5–7^ The data reported herein suggest that ACM is still a major cause of SCD in competitive athletes but with a clear shift toward the LV variant. While classical RV or BIV ACM phenotypes are often suspected because of ECG abnormalities, variants with predominant or isolated LV involvement are mostly characterized by normal ECG and negative 2D echocardiography.^12–14^ Noteworthy, RV aneurysms, which have been considered pathognomonic of RV ACM, were detected only in a minority of cases (20%) in our series, and never in athletes who died suddenly after 2010. The only manifestations may be exercise-induced VAs, so that maximal exercise testing may increase the diagnostic yield of the PPS.^23^

A subanalysis of data by study period (before the first ARVC diagnostic criteria in 1994, between 1995 and 2010 with the introduction of the updated diagnostic criteria, and after 2010) demonstrated a progressive disappearance of cases dying suddenly with T-wave abnormalities and an increased prevalence of a normal 12-lead ECG.

Instead, LQRSV on 12-lead ECG were present in one fourth of competitive athletes with ACM, without statistically significant differences across the study periods. In healthy individuals and athletes, LQRSV have been reported to be rare (2.2% to 4% of elite athletes, 0.5% of recreational athletes, and 0.3% of sedentary individuals) but have recently been described as associated with ACM.^24^

Furthermore, when performed, maximal exercise testing was able to elicit left bundle-branch block or polymorphic VAs in more than half of cases. Thus, our data confirm the indication that LQRSV and exercise-induced VAs should raise the suspicion of an underlying ACM.^25–29^

### Implications for Preparticipation Screening

The fact that a substantial proportion of athletes who died suddenly showed abnormalities at PPS may reflect either the absence of a definitive diagnosis despite abnormal clinical findings or an underestimation of the associated arrhythmic risk at the time of fitness-to-play assessment. In particular, in athletes with ECG abnormalities or VAs, a normal echocardiogram was likely considered sufficient to exclude structural heart disease.

Instead, we confirmed previous studies showing the low sensitivity of 2D echocardiography in athletes with ACM, particularly the LV variant.^4,12,28,29^ Two-dimensional echocardiography data were available in 21 cases, including 4 athletes who were disqualified from sports activity, with normal findings in all except 1 case. In contrast, CE-CMR was performed in 5 of them and consistently yielded positive findings.

Compared with echocardiography, CE-CMR provides superior spatial resolution and tissue characterization, enabling the detection of LGE, which reflects myocardial fibrosis and scarring—key markers of an arrhythmogenic substrate.^30^ While echocardiography remains a valuable first-line tool due to its availability and cost-effectiveness, our study suggests that a high index of suspicion is required in athletes with PPS abnormalities suggestive of ACM, particularly specific 12-lead ECG changes such as LQRSV and VAs during exercise. These findings should prompt second-line investigations, such as 2D-echocardiography, exercise testing and 24-hour ambulatory ECG monitoring, including a training session. Although CE-CMR may not be indicated in all athletes with LQRSV or premature ventricular complexes, it should be performed when additional red flags are present (positive family history, symptoms, other abnormal/borderline ECG findings, or VAs on exercise testing/Holter monitoring), even if echocardiography is normal.^12,31^

### LV Scar and Recommendations for Sport Activity

International guidelines still consider the so-called nonischemic LV scar within the broader context of myocarditis,^32^ while it is also the pathological hallmark of LV-ACM. According to the ESC recommendations, athletes with LGE after myocarditis may return to competitive activities if they have no LV dysfunction or frequent/complex VAs, but they should remain under close clinical monitoring. Our data instead support the current national Cardiological Guidelines for Competitive Sports Eligibility recommendations.^15^ According to these guidelines, the presence of LGE in more than 3 myocardial segments of the LV free wall is a strong contraindication to competitive sports participation, regardless of the presence or absence of symptoms or VAs. This is due to the well-established correlation between the extent of myocardial fibrosis and the risk of life-threatening VAs. Therefore, the presence of multisegmental LGE on CE-CMR should prompt immediate restriction from competitive sports and initiation of a structured clinical follow-up, with consideration of Implantable Cardioverter Defibrillator therapy depending on individual risk factors.

Notably, the athlete in our series with misdiagnosed previous myocarditis highlights the potential dangers of allowing continued physical exertion in athletes with suspected ACM before diagnostic clarity is achieved. Moreover, it illustrates how even an advanced successful interventional treatment, such as the ablation with elimination of inducible RV outflow tract tachycardia, does not cancel the arrhythmic risk when the underlying substrate is mischaracterized.

### Secondary Prevention of SCD in Athletes

Regrettably, within the cohort presented, an AED was available in only 3 out of 24 SCD events occurring during competition (12.5%). Indeed, the Balduzzi Decree, which establishes the mandatory presence of an AED in sports facilities, was approved in 2012, but its enforcement became effective, making compliance obligatory only in 2017.^33^ For these reasons, it is not possible to determine whether on-field AED availability would have conferred a survival benefit for athletes affected by ACM.

### Male Sex Is Predominant in SCD in Athletes

Our data confirm a striking sex disparity in SCD among athletes,^34^ since only 1 of the 51 cases of SCD attributable to ACM, occurred in a female athlete. This finding aligns with and reinforces previous literature suggesting a significantly lower incidence of ACM-related SCD in female athletes compared with males.^35^ There are several possible explanations for this imbalance. Male athletes tend to participate more frequently in high-intensity and endurance sports, which are known to increase arrhythmic risk in individuals with underlying ACM.

### ACM in Athletes During Pediatric Age

ACM has always been considered a rare cause of SCD in pediatric and adolescent athletes.^36,37^ In our series, 7 (13.7%) athletes were younger than 18 years. Noteworthy, 2 of them had an early variant of LV ACM with extensive, band-like myocardial inflammatory infiltrates. Therefore, ACM in this age group remains an exceptional finding, but when present, it carries a high risk during intense physical activity, warranting careful consideration in cardiovascular screening programs.

### Temporal Trends in SCD: Athletes Versus Nonathletes

In our study, we included all ARVC/ACM patients aged 12 to 35 years, allowing a comparison between athletes and nonathletes. The updated analysis confirms a low incidence of SCD among athletes, accompanied by a decline in ACM cases in recent years. However, isolated cases of LV-ACM are still observed in both athletes and nonathletes.

We confirm previous findings showing that the relative risk of SCD was 3× higher in the athletic population during the pre-2010 period, whereas this is no longer the case after 2010. It should be noted that while in the pre-2010 period cardiac arrest almost invariably resulted in SCD, more recently cardiac arrest encompasses both SCD and potentially aborted SCD due to secondary prevention with AEDs.

### Limitations of the Study

The incidence rates reported in this study are provided for epidemiological purposes and were not intended to support causal inference regarding the impact of the 2010 diagnostic criteria or changes in screening policies. Because of the observational nature of this study and the small number of events, formal hypothesis testing or causal modeling would be potentially misleading. Therefore, the epidemiological findings should be interpreted descriptively and considered hypothesis-generating at the population level.

### Conclusions

Our prospective 4-decade Registry of SCD in young competitive athletes shows that ACM still plays a major role; however, its prevalence in our series decreased to 22%, and a phenotypic shift toward the LV ACM variant has been observed, accounting for the normal or borderline ECG and echocardiographic findings. Nonetheless, maximal stress test can trigger left bundle-branch block or polymorphic VAs in more than half of cases. A high index of suspicion is needed, and CE-CMR is crucial for early identification of ACM and athlete disqualification to prevent SCD.

## ARTICLE INFORMATION

### Acknowledgments

The authors thank all the forensic and general pathologists who contributed to the autopsy study of sudden cardiac death (SCD) in the Veneto Region, North-East Italy. Drs De Gaspari, Rizzo, and Basso are supported by the Registry for Cardio-Cerebro-Vascular Pathology, Veneto Region, Venice, Italy.

### Disclosures

None.

### Supplemental Material

Genetic Screening Methods and Results

## Supplementary Material
