# Supplementary material for: Sudden Death in Young Competitive Athletes Due to Arrhythmogenic Cardiomyopathy: A 4-Decade National Referral Center Experience
Source: Circ Arrhythm Electrophysiol. 2026 Jun 17;19(7):e014877. doi: 10.1161/CIRCEP.125.014877 (PMC13387692; doi:10.1161/CIRCEP.125.014877)
Supplement: Supplementary file 1 [file hae-19-e014877-s001.pdf]

SUPPLEMENTAL MATERIAL

GENETIC SCREENING

Methods. Each patient underwent genetic testing using either venous blood samples or paraffin embedded tissue. Coding exons and intronic boundaries of 174 genes related to inherited cardiovascular diseases and SCD were captured for each proband by using the Trusight Cardio kit (Illumina, San Diego, California). Sequencing was performed using the Miseq platform (Illumina, San Diego, California) with 2 X 150 base read length following Illumina protocols. Bioinformatics analysis was performed by means of a custom pipeline including software for variant calling, genotyping, and annotation. Mean coverage for all the evaluated genes ranged between 250 and 400. All synonymous and intronic (other than canonical splice sites) variants were excluded. Genetic variants were also interrogated in the 1000 Genomes project (www.1000genomes.org), the Exome Aggregation Consortium (ExAC) (http://exac.broadinstitute.org), and gnomAD databases (http://gnomad.broadinstitute.org/). Predicted functional effect of a coding variant was surveyed using Polyphen-2 (http://genetics.bwh.harvard.edu/pph/), SIFT (http://sift.jcvi.org/), MutationTaster and Combined Annotation Dependent Depletion (CADD) (http://cadd.gs.washington.edu/). The allele frequency threshold to consider a variant clinically relevant was ≤0.02%. Pathogenicity of variants was classified according to current guidelines. Those variants considered clinically relevant were validated and first-degree relatives were evaluated by direct sequencing (ABI3500Dx, Life Technologies).

Results. Due to the limited availability of post mortem genetic testing before 2010, screening was feasible in 36 cases, revealing P/LP gene mutations in 10 (27.7%).

More in details, 5 out of 18 athletes with RV ACM had positive genetic screening (27.7%), with PKP2 gene mutation in two cases, and DSG2+PKP2, DSC2, and DSP gene mutation, one each. Three out of 13 athletes with isolated or dominant LV ACM had positive genetic screening (23%) with DSP, FLNC, and DES gene mutations, respectively. Finally, 2 out of 5 (40%) with an early variant of ACM were positive for DSP and TTN gene mutations.
